# Foliar Silicon Spray before Summer Cutting Propagation Enhances Resistance to Powdery Mildew of Daughter Plants

**DOI:** 10.3390/ijms23073803

**Published:** 2022-03-30

**Authors:** Jie Xiao, Yali Li, Byoung Ryong Jeong

**Affiliations:** 1 Department of Horticulture, Division of Applied Life Science (BK21 Four), Graduate School of Gyeongsang National University, Jinju 52828, Korea; jiexiao0852@gmail.com (J.X.); yalilee1001@gmail.com (Y.L.); 2 Institute of Agriculture and Life Science, Gyeongsang National University, Jinju 52828, Korea; 3 Research Institute of Life Science, Gyeongsang National University, Jinju 52828, Korea

**Keywords:** antioxidant system, disease severity, pathogenesis-related genes, photosynthesis, reactive oxygen species

## Abstract

Silicon (Si) has beneficial effects on not only plant growth but also against biotic and abiotic stresses. However, a few studies focus on how Si application helps strawberry (*Fragaria* × *ananassa* Duch.) resist powdery mildew. The aim of this work was to find out the optimal Si application method before cutting propagation to enhance the resistance to powdery mildew in strawberry “daughter” plants. Naturally infected “mother” plants of ‘Sulhyang’, ‘Maehyang’, and ‘Kuemsil’ strawberries were supplied with Si. Potassium silicate (K_2_SiO_3_) at a final concentration of 75 mg·L^−1^ Si was either added to the medium for drenching or sprayed to the leaves of the “mother” or “daughter” plant, or soluble Si fertilizer was used to dress the “mother” plant. The Si application significantly increased the shoot fresh weight of the “daughter” plants. Supplemental Si also increased the contents of phosphorus (P), potassium (K), and magnesium (Mg). In addition, the Si treatment decreased the damage of powdery mildew by increased level of proline content and suppressive reactive oxygen species. After applying Si, the length and density of hyphae on the leaf surface decreased. In addition, the infected area of “daughter” plant leaves covered with powdery mildew decreased. This study also demonstrated that Si increased the expression of resistance-gene and decreased the expression of susceptibility-gene of strawberry. Overall, Si application promoted the growth of the “daughter” plants regardless of the application method. Direct foliar Si spray to the “daughter” plants before cutting propagation is recommended to increase their resistance to powdery mildew.

## 1. Introduction

Strawberry (*Fragaria* × *ananassa* Duch.) is a major horticultural crop in Korea [1]. These plants are propagated with runners instead of seeds in commercial farms because the asexual propagation by runners maintains unique characteristics and gives greater yields [2]. The axillary buds of “mother” plants start developing into runners in March and gradually grow into “daughter” plants, and growers prepare them by cutting or pinning in June [3]. During this period, high humidity and closed culture systems facilitate rapid growth and spread of diseases, especially powdery mildew (*Podosphaera aphanis* syn. *Sphaerotheca macularis* f. sp. *Fragariae*) [4]. Leaves infected with powdery mildew display white patches of mycelium, experience severely reduced photosynthesis, and eventually die [5].

Strawberries vary in their susceptibility to powdery mildew, and different genes may confer susceptibility (S-gene) or resistance (R-gene) to different diseases [6]. Mildew resistance locus o (MLO) is a seven transmembrane protein and contains the C-terminal calmodulin-binding domain that is required for powdery mildew infection [7]. Therefore, mutants without the gene *MLO* function can resist powdery mildew [8]. Conversely, the R-gene plays a key role in plant defense against pathogens. *Resistance to powdery mildew 8.1* (*RPW8.1*) and *RPW8.2* genes in *Arabidopsis thaliana* confer broad resistance to powdery mildew. The resistance to powdery mildew of the two genes is conferred by the salicylic acid (SA) and enhanced disease susceptibility (EDS1) dependent signaling pathways [9]. Moreover, the R-gene mediates the expression of pathogenesis-related (PR) proteins, including all microbe-induced proteins and their homologs, such as chitinase (belonging to PR3), peroxidase (belonging to PR9), and lipid-transfer proteins (belonging to PR14) [10].

Traditionally, the disease control of strawberry plants depended on fungicides and soil fumigation [11]. However, with the enhancement of fungicide-resistance in fungi along with environmental concerns, researchers have been on the lookout for environmentally safe alternatives or supplements to traditional fungicides [12,13]. Silicon (Si) is the second most abundant element in the earth’s crust, accounting for 28.8% of the total weight [14]. Different plants contain varying Si levels in their shoots; plants that contain less than 0.5% of Si in their shoots are classified as Si excluders, plants that contain between 0.5% and 1.0% of Si in their shoots are classified as Si intermediates, and plants that contain over 1.0% in their shoots are classified as Si accumulators [15]. The difference in the Si accumulation of different plants stems from the ability of the roots to absorb Si. Plants absorb Si from the soil in the form of H_4_SiO_4_, where the Si is transported into the cortical cells from the external solution, and subsequently released to the xylem [16]. The two processes are based on the transport mediate proteins called low silicon rice 1 (Lsi1) and Lsi2; the Lsi1 and Lsi2 are, respectively, localized at the exodermal and endodermal cells of roots, and cooperate to uptake Si from the soil; the rice mutant *lsi2* results in a defect of Si absorption [17]. Then, Si is transferred into the xylem as silicic acid by Lsi6, which is localized in the adaxial side of the xylem parenchyma cells in the leaf sheaths and blades [18]. Lastly, silicic acid is gradually transformed into amorphous silica and deposited inside cells of the leaf epidermis [19]. It remains unclear which species possess adequate Si transport systems. Strawberry is a Si accumulator species, in which *Lsi1* and *Lsi2* were first identified in 2017 [15,20]. These genes provide new insight into optimizing Si absorption in strawberry. Additionally, the application method also influences how Si affects strawberries as its transport in strawberries is unidirectional [3]. Si has benefits on the photosynthesis, growth, and development, and especially on the resistance to abiotic and biotic stresses in strawberry [21,22,23,24,25,26]. It has yet to be clearly determined how silicon affects specific internal mechanisms to increase such stress resistance in strawberry.

Among strawberry cultivars investigated in this study, ‘Sulhyang’ is the most widely planted cultivar with a high yield [27]; ‘Maehyang’ is a highly firm cultivar that was released by Nonsan Strawberry Experiment Station of Chungnam ARES in 2002 [28]; ‘Kuemsil’ was derived as an artificial cross between ‘Sulhyang’ and ‘Maehyang’ and selected in 2011 [29]. Although powdery mildew is one of the most pervasive and serious diseases for strawberry, the assessment of the severity is generally difficult [30]. Carisse [31] built a model to predict powdery mildew in strawberry and suggested that the severity can be estimated according to the number of diseased leaves. However, it is inapplicable for strawberry “daughter” plants because they only possess two or three compound leaves.

The purpose of this work is to find out the optimal Si application method before cutting propagation to enhance the resistance to powdery mildew in strawberry “daughter” plants. We provide a method for estimating the disease severity (score and the area) in strawberry leaves. The growth and powdery mildew occurrence of strawberry “daughter” plants were evaluated after Si application. In addition, the antioxidant enzyme and reactive oxygen species levels were measured. Lastly, we studied the relative expressions of Si transfer, *PR*, and powdery mildew S- and R-genes, to preliminarily dissect the response of resistance to powdery mildew incurred by Si, and to find the optimal method of Si application to promote powdery mildew resistance in strawberry “daughter” plants.

## 2. Results

### 2.1. Growth and Powdery Mildew Severity

After 8 weeks of treatments, the growth parameters of strawberry plants were measured, as shown in Table 1. The shoot fresh weight of “mother” plants increased with the Si treatments, especially when dressed in a soluble Si fertilizer. However, sprayed Si to “daughter” plants did not significantly increase the fresh weight of the “mother” plants. Similarly, strawberry “daughter” plants treated with Si were bigger compared to the control. Regardless of the cultivar, sprayed Si to “daughter” plants resulted in the greatest shoot fresh weight of the “daughter” plants.

**Figure 1:**
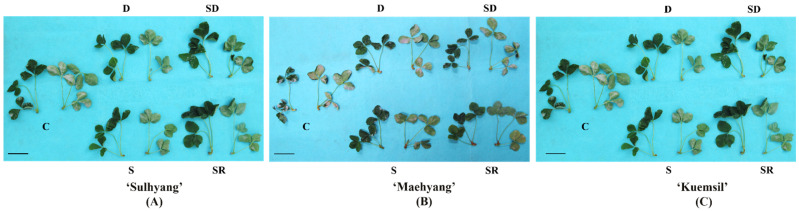
Photographs showing the front and back of leaves of the “daughter” plant (**A**) ‘Sulhyang’; (**B**) ‘Maehyang’; and (**C**) ‘Kuemsil’ infected by the powdery mildew: C, control; S, sprayed “mother” plant; D, drenched “mother” plant; SD, dressed “mother” plant; and SR, sprayed “daughter” plant. Bars indicate 10 cm.

**Figure 2:**
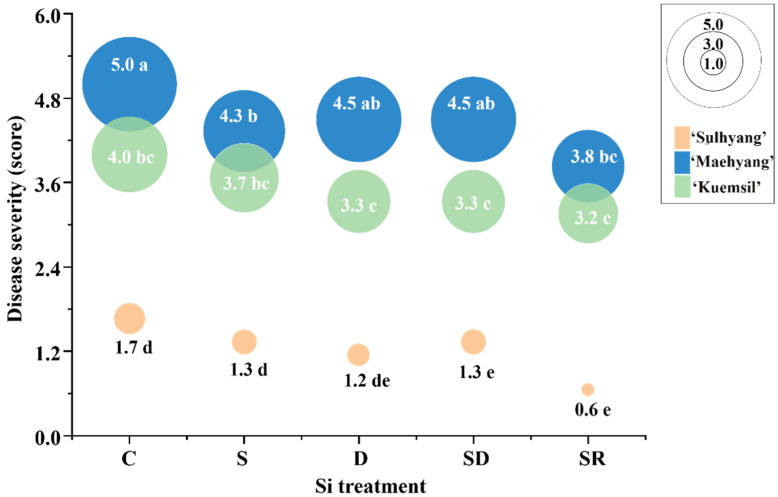
The powdery mildew severity in strawberry “daughter” plants after Si treatments. Lowercase letters indicate significant differences calculated by the Duncan’s multiple range test at *p* ≤ 0.05: C, control; S, sprayed “mother” plant; D, drenched “mother” plant; SD, dressed “mother” plant; and SR, sprayed “daughter” plant.

Varying degrees of white patches were observed on the leaves of strawberry “daughter” plants (Figure 1 and Figure 2). ‘Maehyang’ suffered the most severe among the three cultivars, where the leaves had visibly more white hyphae, and appeared to have shriveled compared to the control (Figure 1B). ‘Kuemsil’ was the second most severely affected by the powdery mildew (Figure 1C), while ‘Sulhyang’ leaves had no obvious white mycelia (Figure 1A). Similarly, the score of powdery mildew severity for ‘Maehyang’ was the highest. Although Si application reduced the powdery mildew severity, ‘Maehyang’ treated with Si had a disease severity score four times that of ‘Sulhyang’ (Figure 2). Thus, the powdery mildew severity was associated with the Si application and strawberry cultivar.

### 2.2. Scanning Electron Microscopy (SEM) and Chlorophyll Fluorescence Characteristics

**Figure 3:**
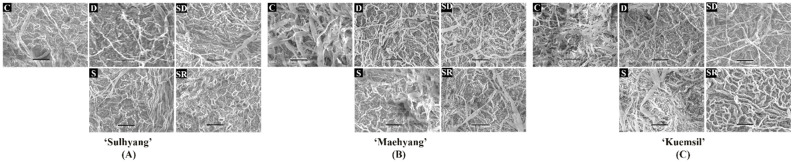
The powdery mildew hyphae on the leaf surface of “daughter” plant (**A**) ‘Sulhyang’; (**B**) ‘Maehyang’; and (**C**) ‘Kuemsil’: C, control; S, sprayed “mother” plant; D, drenched “mother” plant; SD, dressed “mother” plant; and SR, sprayed “daughter” plant. Bars indicate 50 μm.

The disease severity can also be reflected by the condition of the mycelia through SEM (Figure 3). Firstly, SEM observation of Si-treated leaves revealed the change of the hyphae morphology, and the hyphae of the control group were more even and smoother than those treated with Si. Si application inhibited hyphae growth and led to a rope-like structure in the hyphae. In the control group, the hyphae covered a greater area and were dense, and except for ‘Sulhyang’, the stomata were completely covered with hyphae. In contrast, Si application limited the hyphae coverage of leaves, and made the stomata visible on the leaves. Foliar Si sprayed of the “daughter” plants resulted in the greatest folding degree of hyphae and the lowest hyphae coverage.

**Figure 4:**
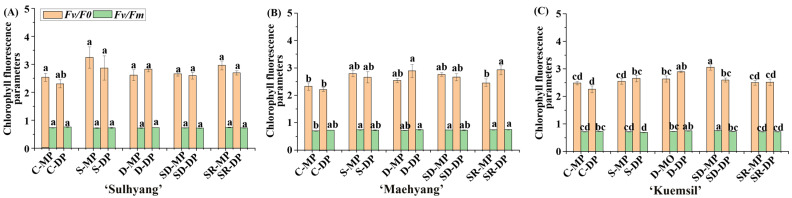
The chlorophyll fluorescence parameters *Fv/Fm* and *Fv/F0* of strawberry (**A**) ‘Sulhyang’; (**B**) ‘Maehyang’; and (**C**) ‘Kuemsil’ as affected by the Si treatments. Lowercase letters indicate significant differences calculated by the Duncan’s multiple range test at *p* ≤ 0.05: C, control; S, sprayed “mother” plant; D, drenched “mother” plant; SD, dressed “mother” plant; SR, sprayed “daughter” plant; MP, “mother” plant; and DP, “daughter” plant.

Hyphae coverage of the leaves also affects their light capture capacity and gas exchange. The stomata are closely related to the chlorophyll fluorescence parameters of the photosystem II (PS II). The chlorophyll fluorescence parameters of the maximum primary yield of PSII photochemistry (*Fv/F0*) and the maximum/potential quantum efficiency of PSII (*Fv/Fm*) are shown in Figure 4. The *Fv/F0* of leaves in the control group and SR-MP decreased except for ‘Sulhyang’, and there were no significant differences in the *Fv/F0* values among Si-treated leaves.

### 2.3. Contents of Micro- and Macro-Nutrients

The macro- and micro-nutrient contents are shown in Table 2. The Si content in strawberry “mother” and “daughter” plants were increased with supplemental Si in the form of foliar spray or medium drench. Furthermore, foliar Si sprayed to the “mother” plants resulted in a higher Si content in “daughter” plants than in “mother” plants. However, sprayed Si on “daughter” plants only increased the Si content in “daughter” plants. Si supplementation also significantly affected the macro- and micro-nutrient contents in strawberry. Si application significantly improved the tissue contents of phosphorus (P), potassium (K), and magnesium (Mg), and the increasing trend of these nutrient levels coincided with the Si supplementation levels.

### 2.4. Hydrogen Peroxide (H_2_O_2_) and Lipid Peroxidation

**Figure 5:**
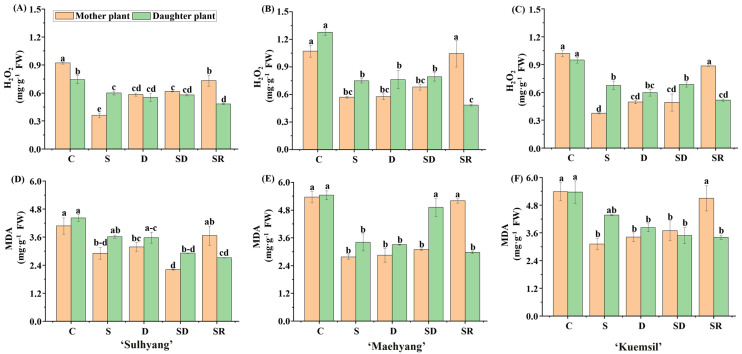
The contents of H_2_O_2_ and MDA of strawberry ‘Sulhyang’ (**A**,**D**); ‘Maehyang’ (**B**,**E**); and ‘Kuemsil’ (**C**,**F**) as affected by the Si treatments. Lowercase letters indicate significant differences calculated by the Duncan’s multiple range test at *p* ≤ 0.05: C, control; S, sprayed “mother” plant; D, drenched “mother” plant; SD, dressed “mother” plant; and SR, sprayed “daughter” plant.

The results of H_2_O_2_ and malondialdehyde (MDA) analyses are shown in Figure 5. The higher levels of H_2_O_2_ and MDA observed in the control indicate cell membrane damage. The cell membrane of ‘Sulhyang’ suffered less damage than that of the other two cultivars. The H_2_O_2_ and MDA contents of Si-treated strawberry, respectively, decreased by 47% and 34% in ‘Sulhyang’, 73% and 46% in ‘Maehyang’, and 56% and 60% in ‘Kuemsil’. This indicated that Si supplementation reduced damages caused by powdery mildew.

### 2.5. Proline Content

**Figure 6:**
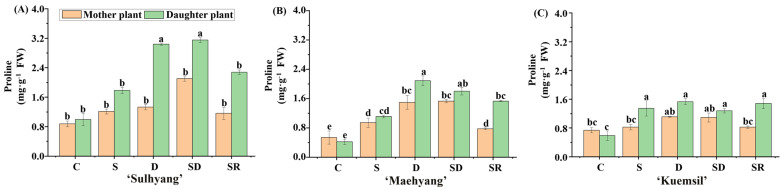
The proline content of strawberry ‘Sulhyang’ (**A**), ‘Maehyang’ (**B**), and ‘Kuemsil’ (**C**) as affected by the Si treatments. Lowercase letters indicate significant differences calculated by the Duncan’s multiple range test at *p* ≤ 0.05: C, control; S, sprayed “mother” plant; D, drenched “mother” plant; SD, dressed “mother” plant; and SR, sprayed “daughter” plant.

The trend of proline content was opposite to that of H_2_O_2_ and MDA contents (Figure 6). In the control leaves, the proline level in ‘Maehyang’ was 50% and 71% of that in ‘Sulhyang’ and ‘Kuemsil’, respectively. The proline accumulation increased in Si-treated strawberry, and the proline levels in ‘Sulhyang’ (2.1 mg·g^−1^), ‘Maehyang’ (1.5 mg·g^−1^), and ‘Kuemsil’ (1.2 mg·g^−1^) were, respectively, 2.1, 2.5, and 1.7 times that in the control (1.1, 0.58, and 0.71 mg·g^−1^). Furthermore, the proline content of ‘Sulhyang’ “daughter” plants treated with Si greatly increased. When “mother” plants were drenched or dressed with soluble Si fertilizers, or when the leaves of “daughter” plants were sprayed, the proline content in the “daughter” plants was 2.0, 2.8, and 1.5 times that in their “mother” plants.

### 2.6. Soluble Sugars and Soluble Proteins

**Figure 7:**
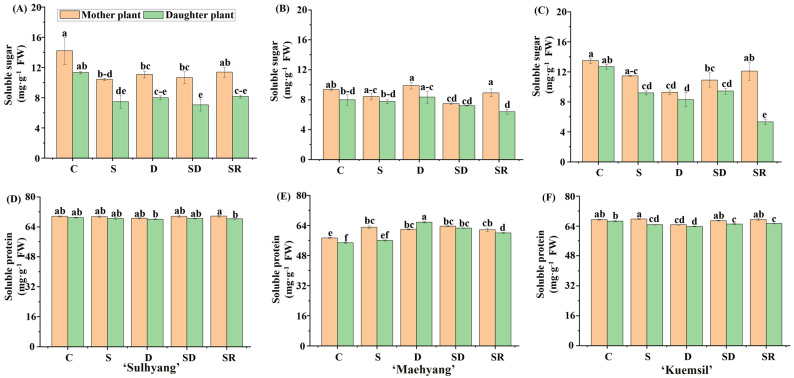
The contents of soluble sugars and proteins of strawberry ‘Sulhyang’ (**A**,**D**), ‘Maehyang’ (**B**,**E**), and ‘Kuemsil’ (**C**,**F**) as affected by the Si treatments. Lowercase letters indicate significant differences calculated by the Duncan’s multiple range test at *p* ≤ 0.05: C, control; S, sprayed “mother” plant; D, drenched “mother” plant; SD, dressed “mother” plant; and SR, sprayed “daughter” plant.

There were significant differences in the soluble sugar contents between the “mother” and “daughter” plants (Figure 7). Among ‘Sulhyang’, ‘Maehyang’, and ‘Kuemsil’, the soluble sugar contents in “mother” plants (11.6, 11.4, and 8.7 mg·g^−1^) were, respectively, 37%, 27%, and 18% higher than that in “daughter” plants (8.4, 8.9, and 7.3 mg·g^−1^). Furthermore, the soluble sugar contents of the control group were slightly higher than that of the Si-treated “mother” and “daughter” plants. Si-treated plants (62 mg·g^−1^) had significantly higher soluble protein contents compared to the control (56 mg·g^−1^) in ‘Maehyang’. For ‘Sulhyang’ and ‘Kuemsil’, no significant differences were observed in the soluble protein content with Si supplementation.

### 2.7. Antioxidant Enzyme Activities

**Figure 8:**
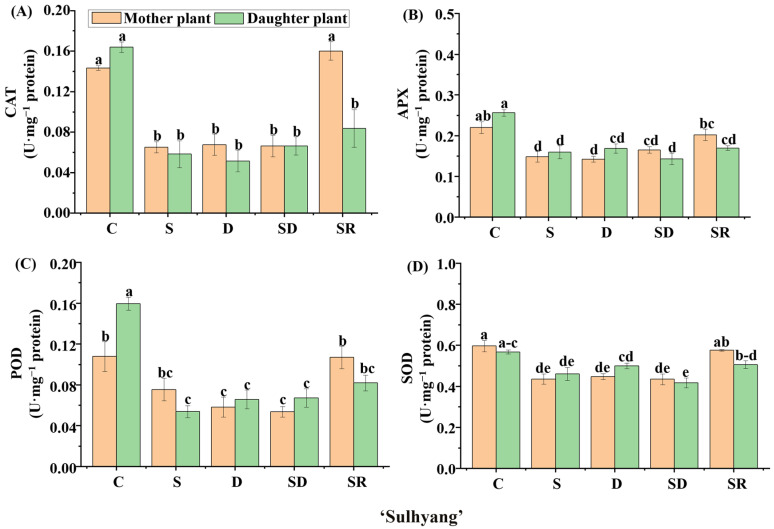
The activities of catalase (CAT) (**A**), ascorbate peroxidase (APX) (**B**), guaiacol peroxidase (POD) (**C**), and superoxide dismutase (SOD) (**D**) in ‘Sulhyang’ as affected by the Si treatments. Lowercase letters indicate significant differences calculated by the Duncan’s multiple range test at *p* ≤ 0.05: C, control; S, sprayed “mother” plant; D, drenched “mother” plant; SD, dressed “mother” plant; and SR, sprayed “daughter” plant.

**Figure 9:**
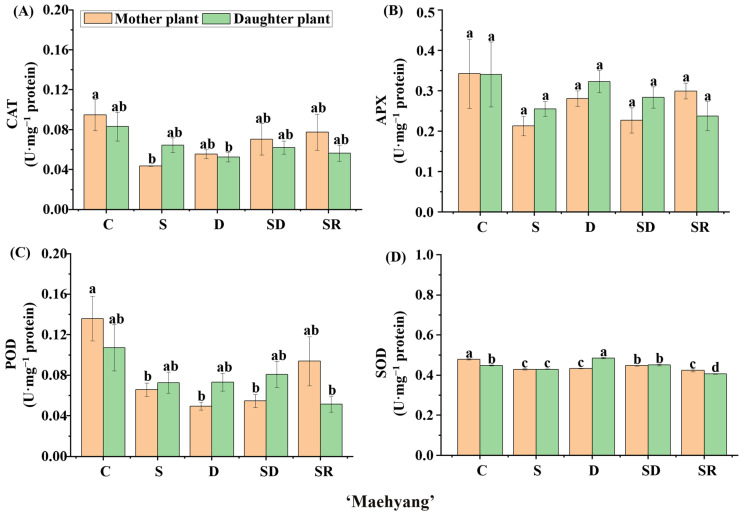
The activities of catalase (CAT) (**A**), ascorbate peroxidase (APX) (**B**), guaiacol peroxidase (POD) (**C**), and superoxide dismutase (SOD) (**D**) in ‘Maehyang’ as affected by the Si treatments. Lowercase letters indicate significant differences calculated by the Duncan’s multiple range test at *p* ≤ 0.05: C, control; S, sprayed “mother” plant; D, drenched “mother” plant; SD, dressed “mother” plant; and SR, sprayed “daughter” plant.

**Figure 10:**
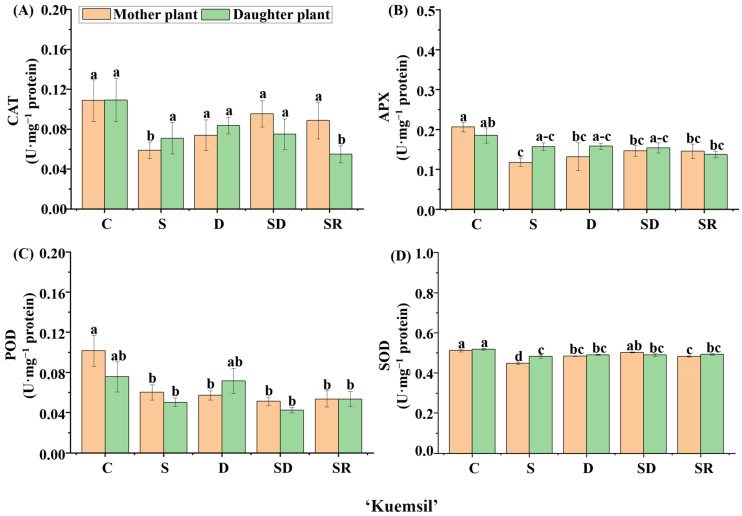
The activities of catalase (CAT) (**A**), ascorbate peroxidase (APX) (**B**), guaiacol peroxidase (POD) (**C**), and superoxide dismutase (SOD) (**D**) in ‘Kuemsil’ as affected by the Si treatments. Lowercase letters indicate significant differences calculated by the Duncan’s multiple range test at *p* ≤ 0.05: C, control; S, sprayed “mother” plant; D, drenched “mother” plant; SD, dressed “mother” plant; and SR, sprayed “daughter” plant.

We further explored the enzymatic activities in ‘Sulhyang’, ‘Maehyang’, and ‘Kuemsil’ leaves (Figure 8, Figure 9 and Figure 10). Significant differences were observed in the catalase (CAT) (Figure 8A, Figure 9A and Figure 10A), ascorbate peroxidase (APX) (Figure 8B, Figure 9B and Figure 10B), guaiacol peroxidase (POD) (Figure 8C, Figure 9C and Figure 10C), and superoxide peroxidase (SOD) (Figure 8D, Figure 9D and Figure 10D) activities. In ‘Sulhyang’, the CAT activities were the greatest without Si application. However, for ‘Maehyang’ and ‘Kuemsil’, Si application resulted in no significant differences in the CAT activities. Similarly, Si application resulted in no significant differences in the SOD activities for all three cultivars. The maximum value of APX (0.34 U·mg^−1^) activities was found in the control ‘Maehyang’ group, although there were no significant differences among the different treatments. Interestingly, Si application slightly decreased the APX activities in both ‘Sulhyang’ and ‘Kuemsil’ which were lower than 0.3 U·mg^−1^. On the other hand, the POD activities were significantly increased with Si application for all three cultivars.

### 2.8. Quantitative Real-Time RT-PCR

**Figure 11:**
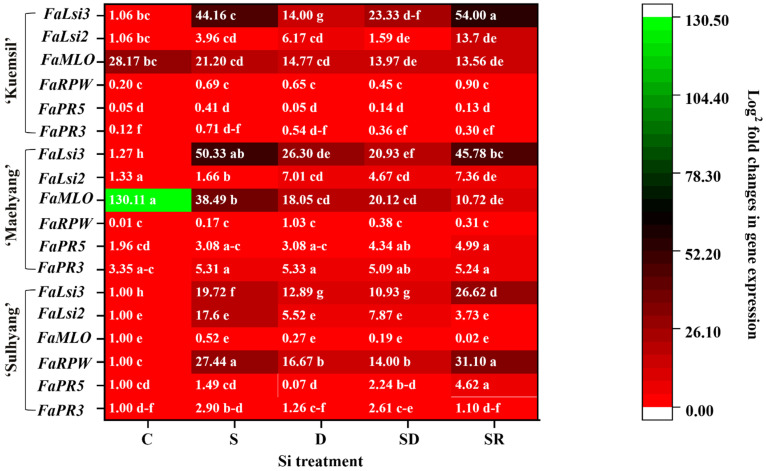
Expression profile heat map of *pathogenesis-related 3* (*PR3*), *PR5*, *resistance to powdery mildew 8* (*RPW8*), *mildew resistance locus o 10* (*MLO10*), *low silicon rice 2* (*Lsi2*) and *Lsi3* in strawberry “daughter” plants ‘Sulhyang’, ‘Maehyang’, and ‘Kuemsil’. The gene expression is presented with a scale of fold change calculated by 2^−ΔΔCT^. Lowercase letters indicate significant differences calculated by the Duncan’s multiple range test at *p* ≤ 0.05: C, control; S, sprayed “mother” plant; D, drenched “mother” plant; SD, dressed “mother” plant; and SR, sprayed “daughter” plant.

The relative expression levels of *FaPR3*, *FaPR5*, *FaRPW8*, *FaMLO10*, *FaLsi2*, and *FaLsi3* showed significant differences in response to the different Si applications (Figure 11). The expression of *FaPR3*, *FaPR5*, *FaLsi2*, and *FaLsi3* were significantly increased in response to Si application. However, the expression of *FaRPW8* was only up-regulated markedly in ‘Sulhyang’ in response to the Si treatments. On the other hand, the expression of *FaMLO10* significantly decreased in response to Si treatments regardless of the cultivar, and the control ‘Maehyang’ had the maximum expression.

## 3. Discussion

### 3.1. Growth and Disease Severity

In this study, the disease severity was assessed according to the area covered by white hyphae. On this basis, the physiological metabolism and expression of related genes were measured. Researchers found that powdery mildew led to premature senescence and reduced the photosynthesis in leaves [32]. This is mainly because the hyphae coverage reduces the functional leaf area and decreases the assimilation rate of the remaining leaf area [33], and limits the leaf gas exchange [34]. In photosynthesis, the CO_2_ transport from the mesophyll cell surface to the sites of carboxylation is a physical process, and interestingly, there is no difference in this process between healthy and diseased leaves [35]. Therefore, the reduced CO_2_ exchange caused by powdery mildew was associated with stomatal closure [33]. In this study, almost all the stomata in the strawberry leaves were closed (Figure 3). However, Si-treated strawberry “daughter” plants had higher *Fv/Fm* values than the control, because Si increased the feed-forward stimulation of the photosynthesis rate instead of affecting the photosynthetic gas exchange [36]. Similar observations were reported in zucchini (*Cucurbita pepo* L.) and melon (*C. melo* L.) [37,38]. Nevertheless, the soluble sugar contents did not increase with the increase in photosynthesis. It is reported that pathogens can absorb nutrients from leaves, especially glucose, as the major carbon energy source [39]. Infection affects the source-sink distribution of plants, which results in an enhanced sugar uptake capacity of the leaves and the activity of sucrose degradation enzymes [40]. In this situation, Si maintains the alteration balance of the sugar production and preserves the source-sink relationship. Similarly, it was reported that Si was beneficial in improving the source-sink relationship between leaves and spikes in rice infected with blast (*Pyricularia oryzae*) by reducing acid invertase activity and content of fructose and glucose [41].

In addition, Si application influenced the nutrient levels in the strawberry plants. It is reported that Si increases P absorption by reducing the soil P sorption [42]; the P content in strawberry tissues increased with Si application, when applied to either the “mother” or “daughter” plants in this study. Our results did not always agree with those of other researchers. In potato, although both soil and foliar Si application increased the Si content in leaves, only soil Si application increased the tissue P content [43]. On the other hand, Si does not affect the soil K and Mg availability [44], but Si application can still increase the K and Mg contents [45,46]. It is likely that Si can affect the uptake, distribution, and status of various nutrients through unknown pathways.

However, the demand of Si fertilizers varies in different agricultural environments [47]. For liquid Si, soil addition was the most effective in alleviating the cadmium (Cd) stresses in wheat [48]. Some researchers on the other hand reported that both soil and foliar Si application are more effective than only soil or spray Si application on their own [49]. In this study, four different Si application methods all increased the growth of “daughter” plants and reduced the disease severity; the greatest resistance to powdery mildew was obtained by spraying the “daughter” plants. Similar results were found on that Si can direct affect the pathogen, and it led to a significant reduction of powdery mildew severity by as much as 80% in wheat plants [50].

### 3.2. Analysis of the Biochemical Mechanisms

There are two opinions on the resistance to biotic stress: From the onset, it was reported that deposition of amorphous silica in the leaves plays a major role in resistance to biological stresses. This sedimentation can form a cuticle-Si double layer to prevent pathogen penetration and to decrease disease incidence [51]. Furthermore, environmental scanning electron microscopy and transmission electron microscopy found that Si led to more Si-papilla on the guard cell of stoma [52,53], which acts as a mechanical barrier against fungal pathogens [54]. Moreover, Si-complexes have been identified in cell walls including hemicellulose, pectin, and lignin, and they accelerate cell wall synthesis and remodeling [55]. The pectinase can’t cleave the C-O-Si bonds of Si-pectin complexes [56]. Therefore, Si maintained mesophyll cells relatively intact under biological stresses. Another suggestion is that Si mediates plant resistance to pathogens through changes in the primary metabolism and molecular aspects, e.g., increasing the contents of chlorogenic acids and activities of antioxidant enzymes [57,58,59,60]. Interestingly, it was reported that the phenomenon of Si accumulation at infection sites is probably due to a higher transpiration rate, rather than serving as an active defensive mechanism [61]. An increasing number of studies focus on the exact nature of how Si interacts with the biochemical pathways leading to disease resistance [62]. In general, SOD defends against reactive oxygen species (ROS) by disproportionation reaction, and APX, CAT, and POD reduce H_2_O_2_ to H_2_O. These enzymes increased in response to mild powdery mildew but decreased in response to severe powdery mildew [63]. However, the antioxidative enzyme activities were greater in seedlings under Cd toxicity, than in seedlings affected by Cd and Si toxicity [64]. Similar results were found in this study, where the Si application increased the POD activity but decreased the APX and CAT activities. It has been reported that POD has a higher tendency to reduce H_2_O_2_ than CAT does [65]. Furthermore, POD also participates in the oxidation of phenolic compounds [66], and Si supplementation can improve stress tolerance by affecting the secondary metabolism [67]. Our study corroborates these findings, demonstrating that Si supplementation significantly increased the proline content. Increasing evidence also showed that Si plays a role in numerous key components in plant signaling systems [68,69]. It was reported that Si treatment contributed to the accumulation of total soluble phenolic and lignin-thioglycolic acid derivatives by increasing the activity of phenylalanine ammonia-lyase (PAL) in banana and coffee plants [70,71]. Moreover, Si stimulated the production of phytoalexins which is critical to defend against pathogen infection in plants [72].

### 3.3. Gene Expressions

To explore how Si affects powdery mildew resistance, it is important to investigate the variation in the genes related to Si transport and strawberry powdery mildew. In this study, there were significant differences in the area of powdery mildew infection among the three cultivars. It is reported that the powdery mildew incidence of ‘Sulhyang’ is lower than that of other cultivars [73]. Our study corroborates this observation with various degrees of powdery mildew severity in the “daughter” plants according to the cultivar. Furthermore, compared to the control, ‘Maehyang’ had the minimum relative expression of *FaRPW8* while that of *FaMLO10* was maximum. This indicates that the ability of ‘Sulhyang’ to tolerate powdery mildew was stronger than that of ‘Maehyang’ and ‘Kuemsil’. Several reports have highlighted the negative regular function of MLO proteins in plant immunity, and that loss-of-function of *MLO* can confer durable and broad-spectrum resistance to diseases [74,75]. Differently, the R product of *RPW* genes acts as a specific receptor of powdery mildew, and activate the subsequent defense responses [76,77].

However, the R-gene is not always expressed, or the resistance is ephemeral because their effects are specific to certain species [78,79]. In this study, Si application increased the expression of *FaRPW8* and simultaneously decreased that of *FaMLO10*. It is reported that Si probably induces the impact of pathogen infection on the transcriptome of host plants, by preventing the virulence of the pathogen [80]. Interestingly, the *FaPR3* expression level in Si-sprayed “daughter” plants of ‘Sulhyang’ only slightly increased, whereas that of *FaPR5* was markedly up-regulated. It is speculated that the resistance of *PR5*-regulate occupies the main position by spraying Si to ‘Sulhyang’ “daughter” plants. Similar results were obtained Si corresponded with *PR1*, *PR2*, and *PR5*, showed higher expression and lower infected area in *Arabidopsis* [81]. Moreover, in this study, the expression of *FaLsi2* slightly increased, while that of *FaLsi3* significantly increased. It is speculated that the Lsi2 protein acted as a Si efflux transporter and was affected by Si influx transporters. Furthermore, in rice, both Lsi2 and Lsi3 were found to be involved in the nodes, but only one efflux transporter, Lsi2, was involved in the roots [18]. Interestingly, the expression of *FaLsi3* was increased by Si spraying on both the “mother” and “daughter” plants, compared to other Si treatments. It is speculated that the Lsi3 protein is probably associated with the Si uptake in leaves.

### 3.4. Application Prospects of Si in Plant Stress

The Si has been demonstrated to benefit growth of rice plants since 1924 [82], and commonly used for improvement of crop production [83]. However, research on the application of Si for plant disease suppression is its infancy. Especially in the context of sustainable agriculture, and greenhouses as an intensive cultivation system, it is necessary to use the environmentally beneficial and non-chemical fungicidal agents [84]. The Si is a crossover element as both fertilizer and plant protection. There is at least 1-year residual effect on disease control of Si applications [85], and more profitable and effective methods of Si application should be explored.

## 4. Materials and Methods

### 4.1. Plant Materials and Estimation of Powdery Mildew Severity

The strawberry “daughter” plants ‘Sulhyang’, ‘Maehyang’, and ‘Kuemsil’ were purchased from a strawberry farm (Sugok-myeon, Jinju, Gyeongsangnam-do, Korea) on 10 January 2021, and planted in a hydroponic gutter system filled with the BVB medium (Bas Van Buuren Substrate, EN-12580, De Lier, Westland, The Netherlands) in a glasshouse at Gyeongsang National University, Jinju, Korea (35°09′ N, 128°05′ E) for two months.

**Figure 12:**
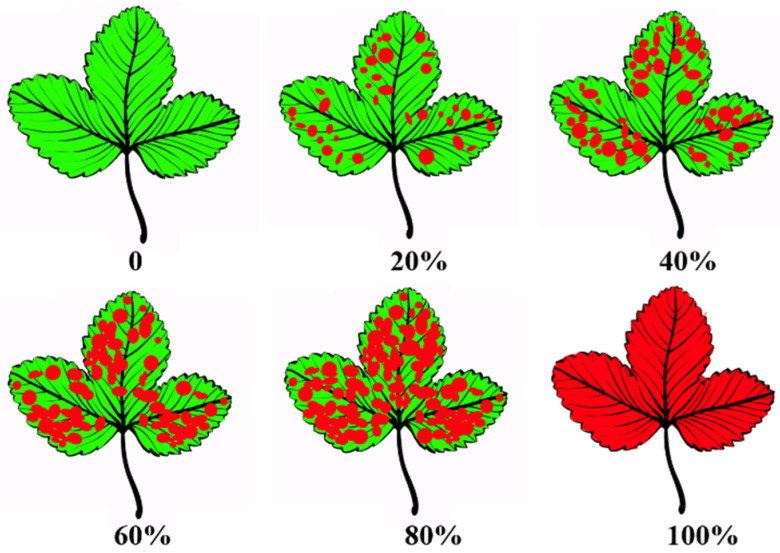
Standard area diagrams for powdery mildew severity on strawberry leaves.

The ‘image processing of conversion’ found in a study [86] was used a reference, and 85,000 pixels were extracted from compound leaves free of powdery mildew infection. The powdery mildew severity was scored from 1 to 5 depending on the number of pixels infected: 17,000 (0~20%), 34,000 (21~40%), 51,000 (41~60%), 68,000 (61~80%), and 85,000 (81~100%) pixels (Figure 12), using the ImageJ graphical analysis software version 1.48v (ImageJ US National Institutes of Health, Bethesda, MD, USA, http://imagej.nih.gov/ij/ accessed on 12 February 2021).

### 4.2. Silicon Treatments

Naturally infected “mother” plants of ‘Sulhyang’, ‘Maehyang’, and ‘Kuemsil’ strawberries with a disease score of 1 were subjected to the Si applications on 10 March 2021. The Si solution was supplied from potassium silicate (K_2_SiO_3_) at a final concentration of 75 mg·L^−1^ was either added to the medium for drenching or sprayed to the leaves of the “mother” or “daughter” plant, or dress 0.5 g of the commercial soluble silicon fertilizer ‘Keunson’ (equivalent to 0.075 g of pure Na_2_SiO_3_) (Saturn Bio Tech Co., Ltd., Gangwon-do, Korea) to the “mother” plant weekly.

### 4.3. Measurement of Growth Parameters

After 8 weeks, the disease severity of the “daughter” plants was estimated and the plant height, crown diameter, chlorophyll fluorescence, chlorophyll level (SPAD), and fresh and dry weights of shoots were examined.

A chlorophyll meter (SPAD-502, Konica Minolta Inc., Osaka, Japan) measured the SPAD. The chlorophyll fluorescence parameters were measured using a portable fluorometer (FluorPen FP110, Photon Systems Instruments, Drásov, Czech Republic). The fresh weight was measured with an electronic scale (EW 220-3NM, Kern and Sohn GmbH., Balingen, Germany). The samples were rinsed with distilled water and dried using a forced air-dry oven (Venticell-222, MMM Medcenter Einrichtungen GmbH., Munich, Germany) at 70 °C for 72 h before the dry weights were recorded. The dried leaves were further used to determine the nutrient contents.

### 4.4. Scanning Electron Microscopy (SEM)

Leaf samples were cut into 0.5 mm^2^ pieces and fixed in 3.0% (*v*/*v*) glutaraldehyde (pH 7.5) for 12 h at 4 °C. Staining was carried out in a 1.0% (*v*/*v*) osmium tetroxide solution for 2 h at 4 °C. The samples were subsequently dehydrated in a graded series of 20, 40, 60, 80, and 100% (*v*/*v*) ethanol, and finally immersed in 80% acetone. The samples were then dried for 2 h at 70 °C and positioned on aluminum stubs with double-stick tape, and gold-coated in a sputter coater (SC7640; Polaron, Sussex, UK). A field emission scanning electron microscope II (SEM/EDS, JSM-7610F, JEOL Ltd., Tokyo, Japan) was used to observe the stomata and mycelium [87].

### 4.5. Determination of Contents of Macro- and Micro-Nutrients

The macro- and micro-nutrient contents were measured according to the method of Jeon [88]. Briefly, 0.5 g dried samples were ashed in a Nabertherm muffle furnace (Model LV 5/11/B180, Lilienthal, Breman, Germany) at 525 °C for 4 h. Then the ash was dissolved in 5 mL 25% (*v*/*v*) HCl, and subsequently diluted with 15 mL of warm distilled water and 10 mL of room-temperature distilled water. The filtrate was finally diluted three times. The macro- and micro-nutrient contents were measured using an inductively coupled plasma (ICP) spectrometer (Optima 4300DV/5300DV, Perkin Elmer, Germany).

### 4.6. Determination of Hydrogen Peroxide (H_2_O_2_) and Lipid Peroxidation

The H_2_O_2_ content was measured according to the method of Junglee [89]. Homogenized 0.1 g leaf samples were extracted in 5 mL 10 mM phosphate buffer (pH 5.8) containing 0.1% (*w*/*v*) trichloroacetic acid (TCA). After centrifugation at 12,000· *g* for 30 min at 4 °C, 5 mL of the supernatant was mixed with 0.5 mL 10 mM phosphate buffer (pH 5.8) and 0.5 mL 1 M Potassium iodide (KI). The absorbance was measured at 350 nm after incubation in the dark for 30 min.

Lipid peroxidation was estimated by measuring the malondialdehyde (MDA) level. Homogenized 0.5 g leaf samples were extracted in 5 mL 0.1% (*w*/*v*) TCA and centrifuged at 10,000· *g* for 5 min. Then 1 mL supernatant was added to 1 mL mixture of 0.6% (*w*/*v*) thiobarbituric acid and 10% (*w*/*v*) TCA. They were then left to react in a boiling water bath for 30 min and rapidly cooled with an ice bath. The absorbance was measured at 450, 532, and 600 nm, and MDA content was determined using the following formula [90]:

$$\mathrm{The}\;\mathrm{MDA}\;\mathrm{content}\;(\mu \mathrm{mol}\cdot g^{-1}\;\mathrm{FW})=6.45\;\times \;\left(A_{532}-A_{600}\right)\;-\;0.56\;\times \;A_{450}$$

### 4.7. Determination of Contents of Proline and Solulel Sugar

The leaf proline content was measured according to Bates et al. [91]. Homogenized 1 g leaf samples were extracted in 10 mL 3% (*w/v*) sulfosalicylic acid. After filtration, 1 mL of the extracting solution was added to 1 mL acid ninhydrin and 1 mL glacial acetic acid, and kept at 95 °C for 1 h in a water bath. Finally, 2 mL cold toluene was added to extract the chromophore and the absorbance was measured at 520 nm.

The soluble sugar content was measured according to the method of Sun [90]. 0.5 g leaf samples were mixed in 10 mL 20 mM phosphate buffer (pH 7.0), then centrifuged at 10,000 rpm for 30 min at 4 °C, then 5 mL H_2_SO_4_ and 1.8 mL distilled H_2_O were added to a 0.2 mL supernatant. Finally, the mixture was kept for 10 min in a boiling water bath, and the absorbance was measured at 620 nm after cooling to room temperature.

### 4.8. Analysis of Contents of Total Soluble Proteins and Activities of Antioxidant Enzymes Activities

The soluble protein content and the activities of superoxide dismutase (SOD), ascorbate peroxidase (APX), catalase (CAT), and peroxidase (POD) were measured according to the established protocols of Soundararajan et al. [92,93]. 0.5 g leaf samples were homogenized with liquid nitrogen and extracted in a 1.5 mL ice-cold 50 mM phosphate buffer (pH 7.0) containing 1 mM ethylenediaminetetraacetic acid (EDTA), 0.05% (*v/v*) Triton X-100, and 1 mM polyvinylpyrrolidone (PVP). The extracts were centrifuged at 13,000 rpm for 20 min at 4 °C, and the supernatant was used immediately to determine the soluble protein contents and activities of antioxidant enzymes.

### 4.9. Quantitative Real-Time PCR Analysis

The CTAB method was adopted for the total RNA extraction [94]. Briefly, 0.1 g frozen leaf tissues were homogenized with liquid nitrogen and mixed with 0.6 mL CTAB extraction buffer, then kept in a 65 °C water bath for 30 min. The supernatant was extracted three times with chloroform after centrifugation (13,000 rpm for 20 min at 4 °C). Afterwards, an equal volume of 6 M lithium chloride (LiCl) was added to the mixture at 4 °C for 75 min. The supernatant was discarded, and the pellet was washed three times in 1.0 mL 75% (*v*/*v*) ethanol. Finally, the pellets were air-dried and dissolved in 20 μL RNase-free diethyl pyrocarbonate water. The quality of the RNAs was determined with the NanoDrop 2000C Spectrophotometer (Thermo Fisher Scientific, Waltham, MA, USA), then reverse transcribed to cDNA using the PrimeScript RT Reagent Kit (Takara, Shiga, Japan). A total of 20 µL reaction volume was constructed with 2 µL each of forward and reverse primers, 2 µL cDNA, 4 µL of RNase-free water, and 10 µL SYBR green.

The *FaActin* was selected as the housekeeping gene. All primers used in this study are shown in Table 3. The CDS sequences were found at Strawberry GARDEN: http://strawberry-garden.kazusa.or.jp/index.html (accessed on 4 February 2022). The running procedure was set to 95 °C for 3 min, followed by 40 cycles of 95 °C for 30 s and 55 °C for 30 s on the CFX96 real-time PCR system (Bio-Rad, Hercules, CA, USA). Three biological replicates were adopted for each treatment.

### 4.10. Data Collection and Analysis

The statistical analysis was carried out using the Statistical Analysis Program (SAS 9.1, SAS Institute Inc., Cary, NC, USA). The experimental results were subjected to an analysis of variance (ANOVA) (*p* ≤ 0.05) and Duncan’s multiple range test (*p* ≤ 0.05). The F-test was also calculated based on Fisher’s least significant difference test at a threshold of *p* = 0.05. Pearson’s correlation coefficient was calculated with the SPSS 17.0 software (SPSS Inc., Chicago, IL, USA). Graphing was performed with the OriginPro software (version 9.0). The relative expression levels were calculated using the 2^−∆∆Ct^ method and using the control as a reference (value = 1).

## 5. Conclusions

Our results showed that the growth and disease severity of strawberry plants were affected by supplementary Si. As expected, powdery mildew inhibited the photosynthesis, and the sensitivity to powdery mildew of ‘Maehyang’ was higher than that of ‘Sulhyang’ and ‘Kuemsil’. Si application promoted the contents of K, P, and Mg, and increased the biomass of the “daughter” plants. Moreover, supplemental Si reduced the contents of H_2_O_2_ and MDA, decreased the cell damage, and increased the resistance to powdery mildew. Among all the Si treatments studied, direct foliar Si spray to the “daughter” plants before cutting propagation was found to be the most effective in promoting their resistance to powdery mildew.

## Figures and Tables

**Table 1:** The growth parameters of strawberry plants under different Si treatments.

Cultivar (A)	Treatment (B)	“Mother” Plant						“Daughter” Plant					
		Plant Height (cm)	Crown Diameter (mm)	Fresh Weight (g)	Leaf Length (cm)	Leaf Width (cm)	SPAD	Plant Height (cm)	Crown Diameter (mm)	Fresh Weight (g)	Leaf Length (cm)	Leaf Width (cm)	SPAD
‘Sulhyang’	C	44.55 de ^z^	18.84 e–g	66.01 c	11.00 d	7.63 c–e	39.55 d	21.52 f	6.06 h	5.12 f	5.65 g	4.7 bc	44.23 ab
	S	46.62 a–d	21.85 b–d	107.04 ab	13.27 a	10.78 a	46.57 ab	26.15 c–e	7.60 e–g	9.95 b–d	6.75 c–f	5.3 a–c	45.52 ab
	D	48.03 a–c	22.14 b–d	90.44 a–c	13.07 ab	10.97 a	45.12 ab	29.53 ab	7.51 fg	11.83 ab	7.43 bc	5.5 ab	37.40 c
	SD	45.05 c–e	23.11 bc	116.24 ab	11.98 a–d	9.20 b	46.43 ab	28.05 a–d	8.18 d–f	11.35 ab	7.58 ab	5.5 ab	39.47 a–c
	SR	44.73 de	19.39 d–g	66.85 b	11.57 b–d	7.97 b–e	46.05 ab	26.30 b–e	9.80 ab	13.99 a	7.18 b–d	5.1 a–c	43.03 a–c
‘Maehyang’	C	43.77 de	17.15 g	63.09 c	11.40 cd	7.15 e	40.48 cd	24.73 d–f	6.67 gh	5.86 ef	6.35 ef	4.3 c	38.88 bc
	S	45.85 b–e	21.18 c–e	86.72 bc	12.53 a–d	7.97 b–e	43.18 a–c	27.20 b–d	6.88 gh	5.81 ef	7.40 bc	6.0 a	42.32 a–c
	D	45.88 b–e	20.54 c–f	82.47 bc	12.93 a–c	8.45 b–d	44.35 ab	24.65 d–f	7.19 fg	9.94 b–d	6.47 d–f	4.6 bc	42.68 a–c
	SD	44.37 de	21.32 c–e	105.33 ab	12.33 a–d	8.78 bc	43.75 a–c	28.77 a–c	8.60 c–e	10.62 a–c	7.60 ab	5.3 a–c	42.28 a–c
	SR	42.87 e	17.96 fg	67.18 c	11.97 a–d	7.32 de	44.28 ab	25.68 c–e	8.57 c–e	11.88 ab	7.55 ab	5.2 a–c	45.03 ab
‘Kuemsil’	C	44.47 de	18.07 fg	94.82 a–c	11.17 d	7.90 b–e	45.33 ab	25.55 c–e	7.08 g	6.80 c–f	6.93 b–e	5.3 a–c	45.90 a
	S	48.45 ab	24.29 b	121.28 a	11.70 a–d	8.03 b–e	45.05 ab	22.90 ef	8.99 b–d	6.48 d–f	6.12 fg	4.7 bc	42.83 a–c
	D	49.80 a	26.93 a	115.25 ab	11.82 a–d	8.47 b–d	43.63 a–c	25.95 c–e	9.65 ab	9.33 b–e	7.20 b–d	5.4 ab	39.23 a–c
	SD	49.38 a	21.90 b–d	121.33 ab	12.53 a–d	8.58 b–d	47.03 a	24.68 d–f	10.11 a	8.22 b–f	6.60 d–f	5.0 bc	41.60 a–c
	SR	48.60 ab	20.20 d–f	93.56 a–c	12.18 a–d	8.22 b–e	42.80 b–d	30.75 a	9.41 a–c	9.38 b–e	8.18 a	6.0 a	44.55 ab
F-test ^y^	A	***	***	***	NS	***	NS	NS	***	NS	NS	NS	NS
	B	***	***	***	*	***	***	***	***	***	***	**	NS
	A × B	***	***	***	***	***	***	***	***	***	***	***	***

^z^ Lowercase letters indicate significant differences calculated by the Duncan’s multiple range test at *p* ≤ 0.05; ^y^ NS, *, **, and *** represent non-significant or significant at *p* ≤ 0.05, 0.01, and 0.001, respectively: C, control; S, sprayed “mother” plant; D, drenched “mother” plant; SD, dressed “mother” plant; and SR, sprayed “daughter” plant.

**Table 2:** Mineral contents in strawberry plants as affected by different Si treatments.

Cultivar (A)	Treatment (B)	Tissue	Si (mg·g^−1^ DW)	Macro-Nutrient					Micro-Nutrient			
				Ca (g·g^−1^ DW)	K (g·g^−1^ DW)	P (mg·g^−1^ DW)	Mg (mg·g^−1^ DW)	S (mg·g^−1^ DW)	Fe (mg·g^−1^ DW)	Mn (mg·g^−1^ DW)	Zn (mg·g^−1^ DW)	Cu (mg·g^−1^ DW)
‘Sulhyang’	C	“Mother” plant	1.15 j–l ^z^	0.18 b–d	0.10 gh	14.99 d–i	20.53 k–m	1.97 i–m	0.75 e–j	0.69 i–l	0.17 b–f	0.10 e–g
		“Daughter” plant	0.83 l	0.08 ij	0.10 f–h	12.18 ij	18.53 m	1.80 j–m	0.66 h–k	0.41 mn	0.14 e–j	0.11 d–f
	S	“Mother” plant	1.65 f–k	0.15 c–f	0.12 c–g	16.56 a–h	29.06 b–g	2.18 h–l	0.65 h–k	0.47 l–n	0.16 b–h	0.12 a–c
		“Daughter” plant	1.69 f–j	0.10 g–j	0.15 a–d	17.34 a–f	19.53 lm	1.81 j–m	0.85 c–g	0.67 i–l	0.10 j	0.10 e–g
	D	“Mother” plant	2.08 d–h	0.16 c–f	0.17 ab	20.19 a	28.03 b–h	3.09 c–g	0.90 b–e	0.72 i–l	0.20 ab	0.12 a–c
		“Daughter” plant	1.92 e–i	0.10 g–j	0.12 c–h	14.63 e–i	20.24 k–m	2.94 c–h	0.65 h–k	0.56 j–n	0.12 h–j	0.10 e–g
	SD	“Mother” plant	1.71 f–j	0.11 g–i	0.12 c–g	15.01 d–i	20.72 j–m	1.14 m	0.62 i–l	0.51 k–n	0.12 h–j	0.10 f–h
		“Daughter” plant	1.16 j–l	0.15 d–f	0.10 f–h	13.63 e–j	25.93 c–k	2.12 h–l	0.92 b–e	0.60 j–n	0.13 f–j	0.12 ab
	SR	“Mother” plant	1.92 e–i	0.10 g–j	0.12 c–h	14.63 e–i	20.24 k–m	2.94 c–h	0.65 h–k	0.56 j–n	0.12 h–j	0.10 e–g
		“Daughter” plant	2.24 d–g	0.11 g–i	0.13 c–f	15.03 d–i	21.65 i–m	1.83 j–m	0.72 f–j	0.77 h–j	0.13 f–j	0.12 a–c
‘Maehyang’	C	“Mother” plant	1.38 h–l	0.13 f–h	0.10 f–h	10.41 j	25.11 d–l	2.70 d–i	0.59 j–l	0.75 h–k	0.12 g–j	0.10 e–g
		“Daughter” plant	0.97 kl	0.11 g–i	0.09 h	16.75 a–g	23.57 f–m	3.47 b–d	0.71 f–j	0.87 g–i	0.18 b–e	0.11 c–e
	S	“Mother” plant	1.63 f–k	0.18 b–d	0.13 b–f	12.37 h–j	27.59 b–h	2.58 e–k	0.66 h–k	1.28 c–e	0.13 g–j	0.09 gh
		“Daughter” plant	1.78 f–j	0.13 e–g	0.14 a–e	17.86 a–e	29.68 b–e	4.16 b	0.62 i–l	1.07 e–g	0.19 a–c	0.09 hi
	D	“Mother” plant	2.55 c–e	0.22 a	0.17 a	14.72 e–i	30.76 a–d	3.33 b–e	0.78 d–i	1.70 b	0.13 f–j	0.11 c–e
		“Daughter” plant	1.77 f–j	0.13 e–g	0.15 a–c	19.09 a–d	36.21 a	3.69 bc	0.70 g–j	0.98 f–h	0.18 a–d	0.10 f–h
	SD	“Mother” plant	2.28 d–f	0.15 d–f	0.14 b–e	12.99 g–j	29.25 b–f	2.18 h–l	0.88 b–f	1.34 cd	0.13 g–j	0.13 a
		“Daughter” plant	1.63 f–k	0.10 g–j	0.16 ab	19.22 a–d	27.25 b–i	4.94 a	0.59 j–l	0.80 h–j	0.22 a	0.12 b–d
	SR	“Mother” plant	1.52 g–l	0.21 ab	0.10 f–h	13.48 f–j	31.55 a–c	2.34 f–l	0.94 b–d	1.99 a	0.11 ij	0.10 e–g
		“Daughter” plant	3.10 c	0.11 g–i	0.14 a–e	19.49 a–c	22.88 h–m	2.64 d–k	0.47 l	0.99 f–h	0.15 c–h	0.03 k
‘Kuemsil’	C	“Mother” plant	1.20 i–l	0.18 a–d	0.11 d–h	15.77 b–i	26.46 c–j	2.93 c–h	1.05 b	1.67 b	0.15 c–h	0.12 ab
		“Daughter” plant	0.95 kl	0.07 j	0.10 f–h	14.73 e–i	18.56 m	3.15 c–f	0.50 kl	0.37 n	0.18 a–d	0.04 k
	S	“Mother” plant	1.70 f–j	0.19 a–c	0.12 c–h	16.74 a–g	28.40 b–h	2.68 d–j	0.90 b–e	1.27 c–e	0.15 c–h	0.12 a–c
		“Daughter” plant	1.83 f–j	0.09 h–j	0.13 c–g	16.49 a–h	20.20 k–m	3.38 b–e	0.63 i–l	0.65 i–m	0.18 a–d	0.03 k
	D	“Mother” plant	3.87 b	0.17 cd	0.12 c–h	19.08 a–d	35.54 ab	2.24 g–l	1.32 a	1.49 bc	0.12 h–j	0.11 b–d
		“Daughter” plant	2.73 cd	0.13 f–h	0.13 c–g	16.84 a–g	20.06 k–m	3.70 bc	0.82 c–h	0.48 l–n	0.13 f–j	0.04 k
	SD	“Mother” plant	2.94 c	0.16 c–f	0.13 c–f	16.16 a–i	30.87 a–d	2.80 d–i	1.20 a	1.48 bc	0.16 b–g	0.11 b–d
		“Daughter” plant	2.07 d–h	0.08 ij	0.12 c–h	19.59 ab	23.19 g–m	3.79 bc	0.96 bc	0.66 i–m	0.20 ab	0.05 j
	SR	“Mother” plant	1.62 f–k	0.18 b–d	0.11 e–h	15.26 c–i	20.31 k–m	4.07 b	0.66 h–k	1.66 b	0.16 b–h	0.09 hi
		“Daughter” plant	4.94 a	0.11 g–i	0.13 c–g	19.08 a–d	24.19 e–m	1.79 k–m	0.50 kl	1.15 d–f	0.14 d–i	0.03 k
F-test ^y^	A		***	**	***	**	***	**	***	***	***	***
	B		***	***	*	***	***	***	***	***	***	***
	A × B		***	*	NS	***	***	***	***	***	***	***

^z^ Lowercase letters indicate significant differences calculated by the Duncan’s multiple range test at *p* ≤ 0.05; ^y^ NS, *, **, and *** represent non-significant or significant at *p* ≤ 0.05, 0.01, and 0.001, respectively: C, control; S, sprayed “mother” plant; D, drenched “mother” plant; SD, dressed “mother” plant; SR, sprayed “daughter” plant; MP, “mother” plant; and DP, “daughter” plant.

**Table 3:** List of qPCR primers used in this study.

Gene Name	Forward Primer (5’ to 3’)	Reverse Primer (5’ to 3’)
*FaPR3*	ACAAACCATCAAGCCACGACG	TTGTCCACGCCCACATTCAAGTC
*FaPR5*	AGGTCCAGTGCAGCAATACCTG	GATTGTCGGACCTCTACCTGCA
*FaRPW8*	CTCTACAACCACGAATCGCTCAAC	GCTCATTCGTATGTCTCTCTTCCTG
*FaMLO10*	GATTATTCACCTGGTCGGACATTGG	ATGGTAAGGACAAGGCAACATCGTA
*FaLsi2*	GCTCTTTTCACCAATGACACCTC	GCATTCACACAAACTCCTACAAGC
*FaLsi3*	AACTGTTCTCTTGCTTGGAGGACG	CCCAAACTTGAGATGGCTCCAGAAT
*FaActin*	TTCACGAGACCACCTATAACTC	GCTCATCCTATCAGCGATT

## Data Availability

Not applicable.
